# Transformation of Ground Vibration Signal for Debris-Flow Monitoring and Detection in Alarm Systems

**DOI:** 10.3390/s120404870

**Published:** 2012-04-13

**Authors:** Clàudia Abancó, Marcel Hürlimann, Bruno Fritschi, Christoph Graf, José Moya

**Affiliations:** 1 Department of Geotechnical Engineering and Geosciences, Technical University of Catalonia (UPC), Jordi Girona 1-3 (D2), 08034 Barcelona, Spain; E-Mails: marcel.hurlimann@upc.edu (M.H.); jose.moya@upc.edu (J.M.); 2 Swiss Federal Research Institute WSL, Mountain Hydrology and Mass Movements, Zürcherstrasse 111, 8903 Birmensdorf, Switzerland; E-Mails: bruno.fritschi@wsl.ch (B.F.); christoph.graf@wsl.ch (C.G.)

**Keywords:** debris flow, monitoring, alarm system, geophones, ground vibration signal, impulses

## Abstract

Debris flows are fast mass movements formed by a mix of water and solid materials, which occur in steep torrents, and are a source of high risks for human settlements. Geophones are widely used to detect the ground vibration induced by passing debris flows. However, the recording of geophone signals usually requires storing a huge amount of data, which leads to problems in storage capacity and power consumption. This paper presents a method to transform and simplify the signals measured by geophones. The key input parameter is the ground velocity threshold, which removes the seismic noise that is not related to debris flows. A signal conditioner was developed to implement the transformation and the ground velocity threshold was set by electrical resistors. The signal conditioner was installed at various European monitoring sites to test the method. Results show that data amount and power consumption can be greatly reduced without losing much information on the main features of the debris flows. However, the outcome stresses the importance of choosing a ground vibration threshold, which must be accurately calibrated. The transformation is also suitable to detect other rapid mass movements and to distinguish among different processes, which points to a possible implementation in alarm systems.

## 1. Introduction

Debris flows are fast movements formed by a mixture of water, solids (sand, boulders, gravel and silt) and, on some occasions, woody debris. Their behaviour is similar to that of liquid concrete [1]. Debris flows threaten people and infrastructures in mountainous areas worldwide, as they travel at high velocities (several meters per second) and can generate great damages due to their high impact forces [2].

Several torrents worldwide have been instrumented with different kinds of sensors and for distinct purposes [3]. On one side, monitoring aims to gain knowledge about the flow behavior, while on another, instrumentation seeks to detect the occurrence of events in order to alert the people exposed to the risk. According to the authors' knowledge, right now, in Europe, debris-flow monitoring stations are mostly located in the Alps: Italy [4,5], Austria [6], France [7] and Switzerland [8,9], but also in the Icelandic fjords [10] or the Spanish Pyrenees [11]. There are other stations in China [12], Japan [13,14], Taiwan [15] and USA [16,17], as well as monitoring stations for other types of rapid mass movements, such as lahars [14,18,19], bedload transport [20,21] or avalanches [22] throughout the world.

Geophones are widely used for detection in debris-flow monitoring stations and alarm systems. However, besides instrumentation to monitor flows, they are also used for other types of processes, such as rockfalls [23,24]. They are a type of ground vibration sensor that records the velocity of small ground movements because of the passage of debris flow. The ground vibration is caused by the energy dissipation of the passing debris flow, due to the impacts of solid material against the channel bed or the interaction between grains. Geophones are generally used as triggering sensors to activate other monitoring sensors or for detection in alarm systems. Their main advantages over other types of sensors being, among others, their robustness, low power consumption or the fact that they can be installed at safe distances, protected from the debris-flow destructive effects. The seismic sensors used in debris-flow monitoring and their features are explained in depth in Section 2.1.

The geophone signal data acquisition process and its analysis show the relevant complexities in the field of debris-flow monitoring. On one hand, the characterization of the measured signal requires high frequency ground vibration sampling rates. Usually the power available in the monitoring stations is limited, which makes the installation of PCs and fast processors difficult (PCs can scan and log at high frequencies). On the other hand, it is crucial to define an appropriate level of vibration to distinguish between the seismic noise of the site which can be originated by many other factors (e.g., wind, lighting strikes, human actions), and the vibrations generated by a debris flow. The definition of such a threshold level for ground vibration is a key task in both monitoring and alarm systems, but defining the optimal threshold value at a specific site remains uncertain. It is important to bear in mind the risk of an unintentional activation, especially when dealing with alarm systems.

The main purpose of this paper is to present a method intended to transform the complex ground vibration velocity signal measured by geophones into a simpler signal, which permits one to detect debris flows and identify their main characteristics. The advantages and drawbacks of this new method will be also evaluated against the registration of the original seismic signal by the sensor. Other minor goals focus on the definition of a threshold value, the factors affecting the seismic signals caused by debris flows and how different types of processes might be distinguished.

The paper is structured into three main parts. First, the characteristics of the available ground vibration sensors are presented and evaluated. Second, the basis of the transformation and its implementation into the hardware are described. Additionally, the influence of the threshold is discussed and some advice for its definition is given. Finally, data recorded in three catchments are shown, and the possibilities of the proposed technique are discussed in detail.

## 2. Ground Vibration Sensors for Debris-Flow Monitoring and Detection in Alarm Systems

### 2.1. Types of Sensors

The collision between particles within a moving debris flow and the impact of the boulders against the bedrock generate seismic signals and underground sounds [25,26]. There are several types of sensors used to detect this ground vibration. Seismometers are extremely sensitive, and detect a wide range of frequencies (including low frequencies). They have been used in several test sites, such as the Moscardo torrent in Italy, e.g., [5,25]. Nevertheless, geophones are the most common ground vibration sensor in debris-flow monitoring systems [9,11,25,27], due to their advantages over other kinds of ground vibration sensors (e.g., robustness, low consumption). They measure the velocity of ground motion. Acoustic sensors have also been tested to register the sound generated by the motion of a debris flow. Microphones to register underground sound [28] or infrasonic devices [29,30] are some examples of acoustic sensors used for debris-flow monitoring. According to recent advances the combination of acoustic sensors and seismic sensors increases the detection probability of events [31].

There are different types of geophones which can record 1D or 3D measurements: piezoelectric geophones and moving-coil geophones. In order to apply the method presented in this paper, 1D moving-coil geophones were used. Moving-coil geophones consist of a magnetic moving mass oscillating inside a wire coil, a mechanism that generates an output voltage proportional to the velocity of the ground vibration in the direction of the coil. The data from the geophone (continuous output voltage) is obtained and stored in a CPU memory, by means of a specific data recording system. This aspect will be more extensively described in the following sections.

### 2.2. Factors Influencing the Ground Vibration Record

The geophones or seismometers used for debris-flow monitoring are generally installed outside the wetted area, to avoid them being damaged when an event occurs. Normally they are placed in a protected location next to the channel. Both the amplitude and frequency of the signal measured by the sensors depend on several site-specific factors, especially their placement and assembly. Furthermore, other external elements can affect the vibration signal (e.g., meteorological elements, or human/animal actions). Normally the influence of some external factors is avoided with structures that cover the geophone and by means of information leaflets to avoid vandalism. But, the effects of site-specific influencing factors are still unknown, which leads once again to the problem of accurately defining ground vibration thresholds.

**Figure 1.**
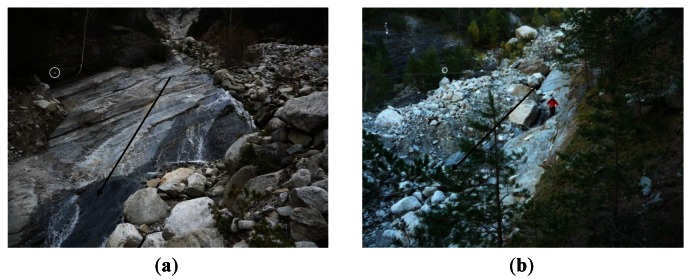
Factors affecting the ground vibration signal: characteristics of transmission medium between geophone and flow path. The transmission medium can be bedrock (**a**) or colluvium (**b**). The location of geophones is indicated by a circle; while the flow direction, is expressed by an arrow.

**Figure 2.**
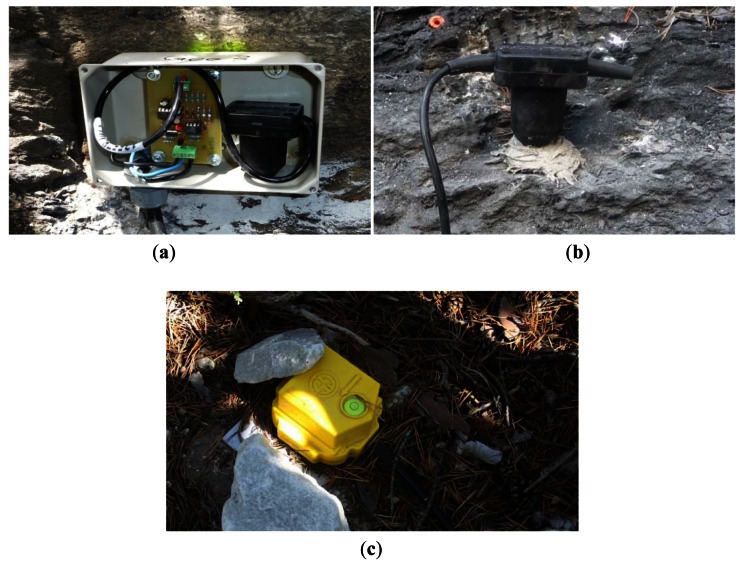
Factors affecting the ground vibration signal: assembly of sensors: (**a**) geophone fixed inside a metal sheet box (a signal conditioner can also be seen in the box), (**b**) fixed directly on the bedrock, and, (**c**) nailed down in the soil. Normally the geophone is additionally protected from the rainfall or hailing.

**Figure 3.**
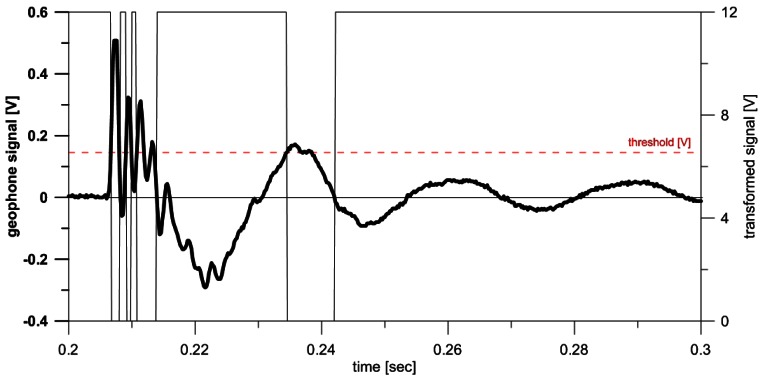
Transformation of the geophone signal (thick line) into a pulse signal (thin line) by the signal conditioner using a previously defined threshold value (dashed red line).

The threshold has to be defined for each individual geophone location. Until now, it was established empirically, following the experience of technicians and researchers [8,17,32]. Therefore, should the effect of the different factors be quantifiable, the process of distinguishing debris flows from seismic noise in the site would be easier and more reliable. There are three main important issues that affect the vibration measured by ground vibration sensors: (1) distance between sensor and the flow path of the debris flow; (2) characteristics of the underground material at sensor location and between sensor location and channel (Figure 1), and (3) type of sensor assembly (Figure 2).

The distance between sensor and flow is key, as the vibration waves are attenuated with the distance [33] and the wave does not travel long distances. It is for this reason that geophones should be installed, at the most, a few tens of meters far from the channel or at its stream banks. Diminishing the vibration signal strongly depends on the physical properties of the transmission medium [28]. For example, P-wave speed ranges from about 350 m/s in alluvium up to 700 m/s in rock [25]. Therefore, if the flow runs over the bedrock and there is no colluvium between the flow and the sensor (Figure 1(a)), the signal does not attenuate as much as in the cases where colluvium is present (Figure 1(b)).

Regarding the assembly of the sensor, two important aspects should be taken into account: (1) the type of material the sensor is located in, and (2) the assembly system. The specifications of the geophones normally limit the assembly angle to a specific value (e.g., 25° in GEOSPACE geophones GS-20DX) with respect to the direction in which the ground vibration is measured. Since the surfaces of assembly are often irregular, different assembly systems are designed in the existing monitoring stations (Figure 2). The assembly structures can show a resilient vibration (Figure 2(a)), therefore affecting or conditioning the signal registered. In contrast, the geophones assembled using either a special structure or directly on the ground (Figure 2(b) and Figure 2(c)) are not thus affected.

### 2.3. Data Recording Systems

Data recording of ground vibration sensors in monitoring stations is normally done by means of an external device (data recording device). The output of the geophone is a continuous voltage proportional to ground velocity, as mentioned above. This voltage can be recorded in different ways, depending on the device used for data recording and the purpose of monitoring. Three different general data recording systems are described below.

First, analog signal recording consists in continuous logging of the voltage measured at the sensor. This technique was applied in the monitoring station of the Moscardo torrent [24], but it is not in use anymore. At Moscardo, a magnetic tape recorder was used, and changed periodically when full.

Second, digital signal recording consists of non-continuous voltage samples from the output signal measured at the geophone [34]. The acquisition device gathers the sample values at a specific frequency. According to the signal processing theories, the sampling rate (*f_s_*) to avoid aliasing problems must be greater than the Nyquist sampling rate, which is twice the highest frequency (*f*_max_) of the signal:

(1) $$f_{s}>2f_{\text{max}}$$

The typical frequencies of the strongest ground vibrations induced by the passage of a debris flow correspond to a range of some 20 to 50 Hz [33]. However, the signal registered by the geophones depends on the characteristics of the geophone, as well as other factors related to the device placement, as mentioned in the previous section. Therefore, the frequency content can diverge from these values. That is why the minimum necessary sampling rate (≥100 Hz) is often a problem, especially when the device used for data recording is a standard datalogger, which normally has a limited sample rate. This type of data recording is thus generally associated with a PC. The problem concerning the use of a PC instead of a standard datalogger is the higher power consumption.

The third type of data recording system is defined as a general case that includes different types of transformations applied to the original ground velocity signal measured by the geophone. To the authors' knowledge, two different transformations of the ground velocity signals were used: (1) the one presented in this paper (transformation into impulses), (2) the one used in the Moscardo Torrent (transformation into amplitude of the velocity signal [25]).

Moreover, the amount of continuous data is very large, and thus, in the field of rapid mass movement monitoring, it is common to have two different recording frequencies: lower frequency (no-event mode) and higher frequency (event mode). For that reason, a trigger is used, defined as an algorithm that checks the variations of the signal that could indicate an event. Defining a reliable trigger for a monitoring system can avoid false alarms that can be caused by factors such as lighting or thunderstorms [11]. The simplest triggers, widely used in seismology, are: (a) level triggers, in which a high frequency recording (event mode) starts whenever the ground velocity threshold is reached; (b) short-term average-long-term average trigger (STA/LTA), which changes from no-event into event when the ratio between STA and LTA exceeds a given threshold. STA is the average of the values of ground velocity in a short term period (typically less than a second or a few seconds) and LTA is the average of ground velocity in a long term period (normally some tenths of seconds) [35]. Other than the simple triggers, more sophisticated ones are also used. They can include different parameters, focusing in amplitude and or frequency of the signal [10].

In this paper, we present a data recording system which consists of transforming the ground velocity signal measured continuously by a geophone (voltage), into a pulse signal (two voltage values). This transformation is useful for data gathering due to its simplicity, as it will be explained in detail in the following sections. This transformation is not specifically associated with a trigger, as mentioned above. However, in those monitoring stations that use this transformation (see following sections), the trigger implemented is a level trigger over a time interval. The level trigger is implemented over the transformed signal [11].

## 3. Transformation of the Geophone Signal into Impulses

### 3.1. The Concept of Transformation of Ground Vibration Velocity into Impulses

The aim of the transformation of the geophone voltage signal is twofold. On one hand, it removes ground vibration noise and external malfunction/disturbing factors. On the other, it converts the continuous signal from the geophone into a simple digital one. The procedure consists of two parts: firstly a signal conditioner transforms the continuous signal into a pulse signal, and then, a counter records the number of impulses. Thus, the resulting transformed signal can be registered by a datalogger with a lower sample rate at a lower consumption, without losing the reliability of detecting the event occurrence and its different phases.

Removing the noise, as well as transforming the original geophone signal, depends on the existence of a threshold value of voltage, which defines the critical ground velocity. This threshold value allows distinguishing between non-desired seismic vibration and the ground velocity induced by debris flows by means of a comparator. The transformed signal has a value of 0 V, if the ground velocity threshold is exceeded in the geophone, or the value of the power voltage (normally 12 V), if the threshold is not exceeded (Figure 3).

The gathering device counts one impulse every time the output signal from the Schmitt trigger (the output of the circuit is retained in the upper value until the threshold is exceeded) [36] changes from the upper voltage to 0 V. The 0 V value of the transformed signal lasts until the geophone signal crosses the line of 0 V. Finally, the counter integrated in the data recording device counts the impulses. In our case, the datalogger saves the number of impulses per second [IMP/s].

### 3.2. The Signal Conditioner

**Figure 4.**
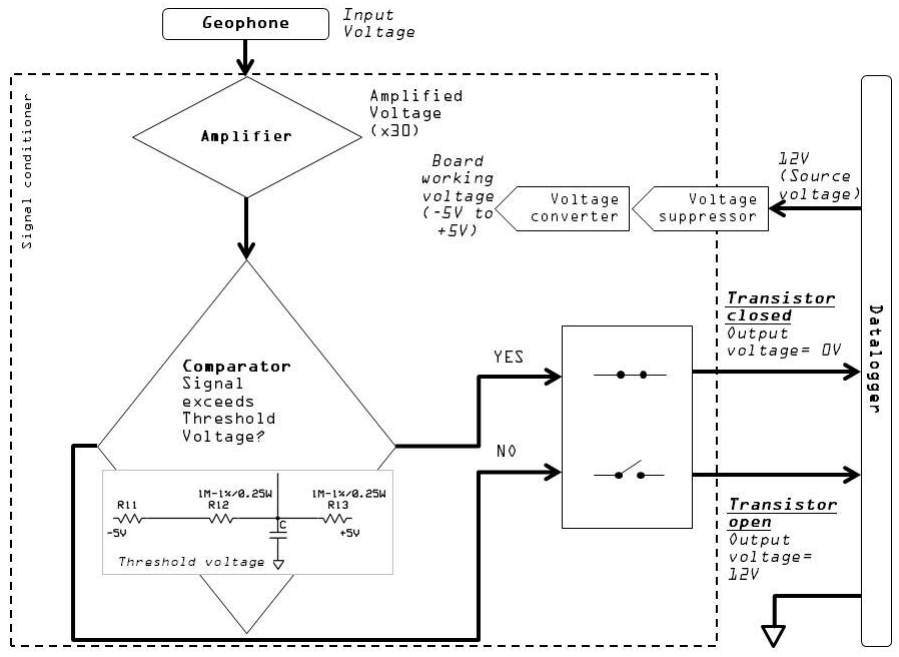
Simplified diagram of the signal conditioner and its interaction with the datalogger and the geophone.

The signal conditioner (Figure 4) consists of a printed circuit board that is connected to the geophone and to the datalogger (see Figure 2(a)). The circuit has a power-consumption below 10 mA, which is provided by the datalogger battery. The power voltage is normally 12 V, because this is the standard voltage source required by the datalogger, and the values of voltages presented in this paper are relative to a 12 V source voltage. However, the system can work with lower voltages.

The transformation of the ground velocity signal into a pulse signal is the main objective of the signal conditioner. This transformation is controlled by the threshold of the vibration, which is set by means of a group of three electrical resistors (R11, R12, R13 in Figure 4). The signal conditioner board has five components: (1) an amplifier, which increases the input signal from the geophone directly at the entrance of the circuit. This amplifier has the function of magnifying the ground velocity signal by a certain factor (in our case: ×30); (2) a comparator, which checks if the voltage exceeds the threshold voltage established by the resistors; (3) a transistor, which regulates the closure of the circuit (threshold exceeded) and consequently the pulse signal value is 0 V or open (threshold not exceeded) and the pulse signal has the value of the power voltage, 12 V; (4) a voltage suppressor, that regulates the input voltage from the datalogger battery and protects the circuit from external factors potentially causing malfunctions; and (5) a voltage converter, that transforms the input voltage coming from the battery (12 V) into the working voltage of the signal conditioner board (−5 V to +5 V).

### 3.3. Selection of the Ground Velocity Threshold

The definition of the threshold is essential for debris flow detection. The threshold should be chosen in such a way that ground vibration induced by seismic noise should be ignored, while the ground vibration caused by the passage of debris flows is preserved. In order to satisfy these two conditions, the definition of the threshold has to be performed at each geophone location taking into account the local influencing factors described in Section 2.2.

**Figure 5.**
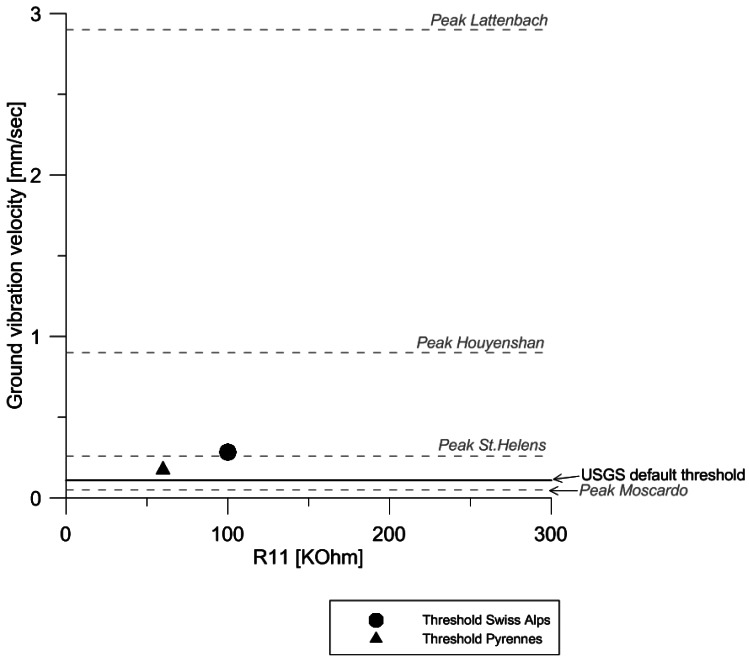
Common threshold values of the monitoring stations in the Pyrenees and the Swiss Alps (indicated by dots), linearly dependent on value R11. Values of ground vibration velocity peaks (dashed grey lines) and thresholds (black continuous line) are given for comparison (see text for detailed explanation on each value).

As mentioned above, the threshold value is controlled by the signal conditioner, in particular electrical resistor R11 (Figure 4). The resistance value of R11 regulates linearly the threshold of ground vibration velocity (Figure 5). The correlation between the resistance value of R11 and the corresponding voltage level can be obtained by applying Ohm's Law. This threshold voltage depends on the value of resistance R11 (the values for R12 and R13 should be fixed at 1 MΩ), and it can be transformed into ground velocity using the transduction constant inherent in the geophone model (e.g., 0.28 V/cm/s in GS-20DX, http://www.geospacelp.com/).

While peak values of ground velocity due to debris-flow occurrence are often found in the literature, threshold values are still rarely published (Figure 5). In Figure 5, the thresholds from the test sites in the Swiss Alps and the Pyrenees are compared with the default threshold value of the USGS Acoustic Flow Monitor [17]. Moreover, some peak values of ground velocity from other monitored sites are included in the plot. In the Mount St. Helens (USA) event of 16 October 2004, the maximum ground velocity was about 0.25 mm/s [33]. In the Moscardo torrent (Italy), the peak value of the ground velocity was 0.05 mm/s during the debris flow occurred in 22 June 1996 [37]. In Houyenshan (Taiwan) the peak value of ground velocity was 0.9 mm/s [38]. In Lattenbach (Austria), the peak value of ground velocity registered reached the 2.9 mm/s [39].

The low peak value of ground velocity from Moscardo can be attributed to the placement of the geophone, buried into the ground and separated about 20 m from the flow path. In general, the peak values of ground velocity exceed the threshold values, as it could be expected. However, it's important to notice that the values of the velocity registered in the geophones are clearly affected by the influencing factors commented in Section 2.2, but also for the magnitude of the event. Therefore, it emphasizes once more the importance of the good adjustment of the threshold value, depending on the placement and the magnitude order of the events occurring in the instrumented torrent.

## 4. Application to Debris-Flow Monitored Sites

### 4.1. Site Description and Data Analyzed

**Figure 6.**
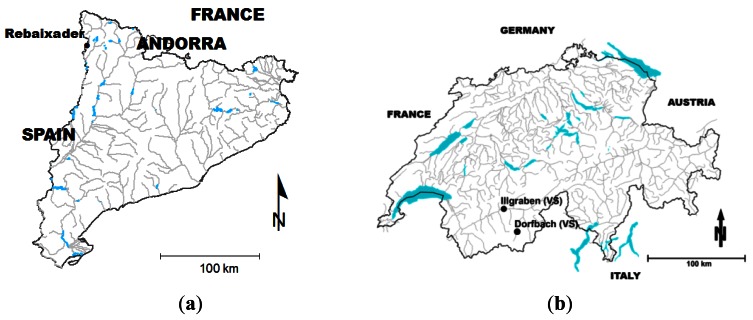
Location of the monitoring stations applying the signal transformation method. (**a**) Catalan Pyrenees (**b**) Swiss Alps.

The signal transformation method proposed in this paper is or was in use in several running or abandoned monitoring stations located in the Pyrenees (P; Figure 6(a)) and the Swiss Alps (SA; Figure 6(b)): Ensija (P), Erill (P), Rebaixader (P), Illgraben (SA), Dorfbach (SA), Preonzo (SA), Riascio (SA) and Schipfenbach (SA). The monitoring systems in the Pyrenees were installed in 2009 [10], and only a few debris flows have been registered until now. In contrast, an extensive database of debris-flow events was gathered in the Swiss Alps during the last decade. In the following sections, data from debris-flow events recorded in the Illgraben and Dorfbach monitoring systems are presented. Moreover, a comparison of the seismic data collected in the Rebaixader torrent shows, how to distinguish between the different processes occurred in the torrent.

#### 4.1.1. Illgraben

**Figure 7.**
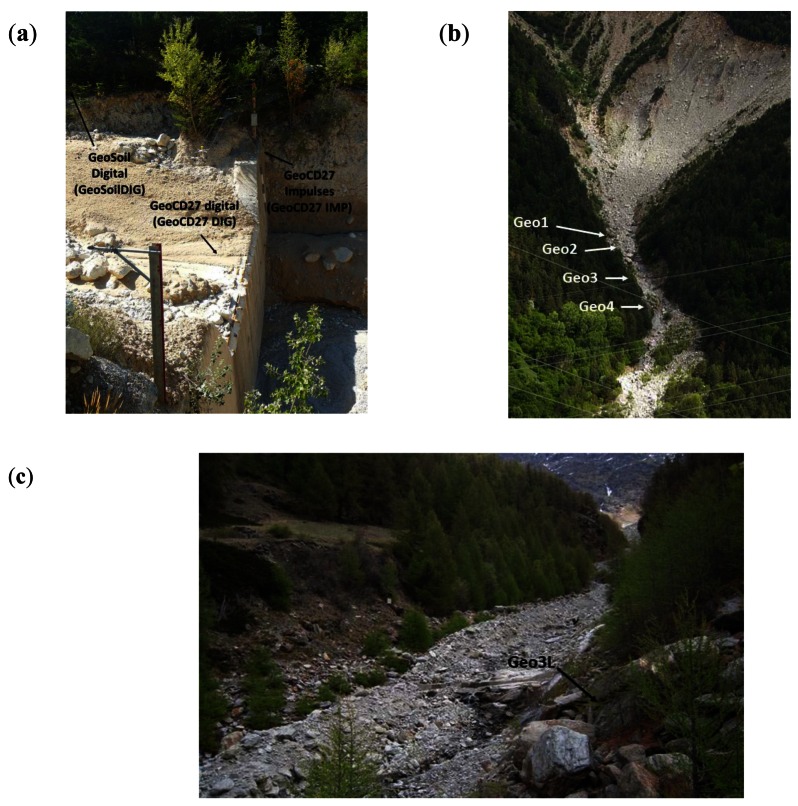
Location of the geophones in the three sites (**a**) Illgraben torrent (Swiss Alps), (**b**) Rebaixader torrent (Central Pyrenees, Spain), (**c**) Dorfbach torrent (Swiss Alps).

The Illgraben catchment (9.5 km^2^) is well-known for its high debris-flow activity and frequent sediment transport [8,9]. It is characterized by steep slopes and a huge amount of sediment originated by the weathered bedrock that forms a large anticline. Debris flows are mainly triggered by high-intensity, short-duration storms [40]. Tens of debris flows, in a wide range of different flow types, were registered by a sophisticated monitoring system. Although many were the types of devices installed, including several types of flow depth sensors, geophones, erosion sensors, *etc.*, only the data recorded at three geophones (located at check dam 27 and its surroundings) were analyzed in this paper (Table 1 and Figure 7(a)). Two data recording systems for the geophone signal were used: (1) digital sampling, as explained in Section 2.3, and (2) signal transformation applying the method proposed. The geophone known as “geoCD27IMP” (fixed on the wall of the check dam, Figure 7(a)), recorded the transformed signal. In contrast, “geoCD27DIG” (buried in the channel bed upstream of the check dam, Figure 7(a)) and “geoSoilDIG” (nailed in the soil, 15 m far from the flow path at the check dam) were connected to a PC and digitized the signal at 2 kHz without applying any transformation. These data were used to compare both data recording systems.

#### 4.1.2. Dorfbach

The Dorfbach catchment (5.7 km^2^) is located in the Mattertal valley, in the Canton of Valais (Switzerland). The Dorfbach has been monitored since 1993 by changing and enlarging the equipment, even though observation was interrupted from 2007 to mid-2010, when a new modernized system was installed. Some small debris flows occurred during the late spring and summer of 2011. These debris-flow events were registered by several devices, which include flow depth sensors and geophones with different thresholds. The geophone signal was gathered by means of signal transformation into IMP/s. In this study, data corresponding to geophone Geo3L and the event in late spring 2011 were analyzed (Table 1 and Figure 7(c)). This geophone signal is transformed by 10 different thresholds in parallel. Each one of these thresholds is controlled by a different electrical resistor.

#### 4.1.3. Rebaixader

The Rebaixader torrent has a catchment area of 0.47 km^2^ and is located near the village of Senet in the Central Pyrenees. The torrent runs over a glacial moraine and bedrock (slates) outcrops. The monitoring system consists of a meteorological station and a flow station. The latter includes one ultrasonic device to record the flow depth, a video-camera and five geophones. Data from four of them were analyzed in this paper applying the signal transformation method presented in this paper (Figure 7(b)). All of these geophones are assembled in a weatherproof box. The distances to the channel and type of material available are given in Table 1.

### 4.2. Comparison between Ground Velocity Signal (GVS) and Impulse per Second (IS) Data

The ground vibration induced by the event occurred in 27 July 2009 in Illgraben was measured by the two geophones located at check dam 27 and a third one placed in the surrounding area (Figure 7(a)). On the one hand, the signal measured by geoCD27DIG and geoSoilDIG was digitized at 2 kHz and stored on a hard disk. The signal measured by geoCD27IMP was transformed by the signal conditioner into impulses using a threshold of 0.71 mm/s. It was stored in a datalogger (Campbell Scientific CR10X) every second.

**Figure 8.**
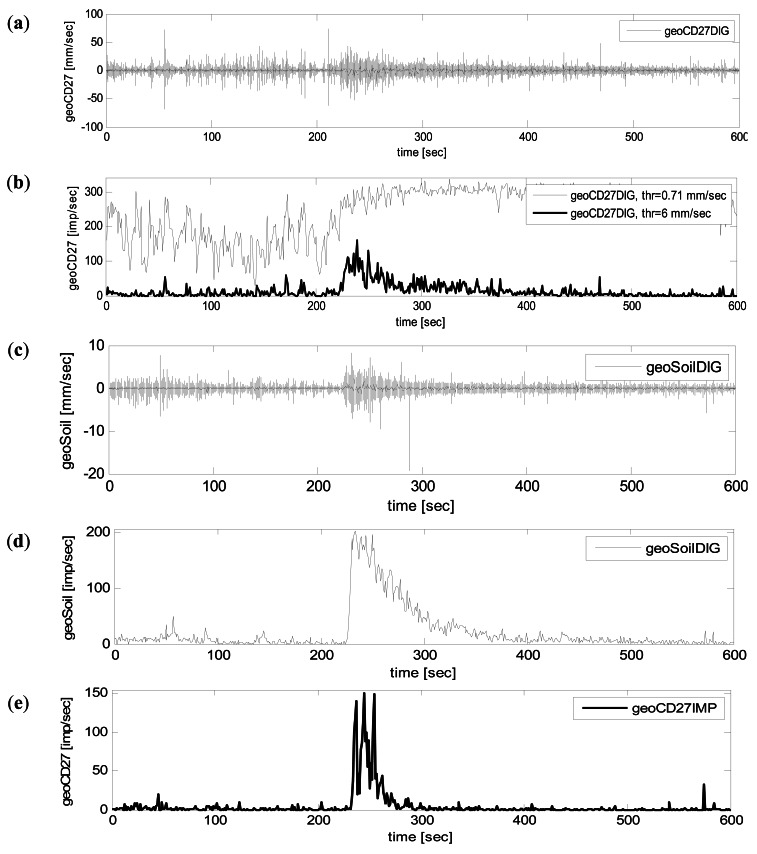
Data recorded in Illgraben during the event occurred in 27 July 2009. (**a**) Ground velocity signal (GVS) measured by geoCD27DIG (sample rate: 2 kHz) (**b**) Impulse per second (IS) time series obtained by post-processing geoCD27DIG data using the MATLAB code (thin line: 0.71 mm/s threshold, thick line: 6 mm/s threshold) (**c**) GVS measured by geoSoilDIG (sample rate: 2 kHz). (**d**) IS time series obtained by post-processing geoSoilDIG data using the MATLAB code (0.71 mm/s threshold) (**e**) IS time series measured at geoCD27IMP applying the transformation by the signal conditioner (0.71 mm/s threshold).

Additionally, a MATLAB 7.9 (released in 2009) code was developed to perform the same transformation as the signal conditioner, but as a post-process. This was done using the original signal of geoCD27DIG and geoSoilDIG, digitized using a PC. Subsequently, it was possible to compare three different time series: (1) ground velocity signal (GVS) recorded digitally at 2 kHz by geoCD27DIG and geoSoilDIG (Figure 8(a) and Figure 8(c)), (2) impulse per second (IS) time series obtained by the MATLAB code from the ground vibration data recorded by geoCD27DIG and geoSoilDIG (Figure 8(b) and Figure 8(d)), (3) IS time series recorded by geoCD27IMP (Figure 8(e)).

Figure 8(a,c) corresponds to GVS time series from each of the two geophones that register the original ground velocity data. In both figures, different stages of the event can be identified. A spindle shape is common in both time series, describing the general trends: (a) Low vibration in the beginning of the time series, which can be attributed to an hyperconcentrated flow stage and the initiation of the debris flow; (b) Sudden increase of ground velocity, which corresponds to the front passage close to the geophone [31]. The amplitude of the ground velocity signal increases as the flow incorporates more mass, mainly by the sediment entrainment [41]; (c) After the passage of the main mass near the geophone, a decreasing can be observed. Additionally, precursory and post-front waves (both smaller than debris-flow front) can be identified [25].

Note that the GVS signal in Figure 8(a) shows high ground velocities, especially compared to values in Figure 5. Although data in Figure 8(a) are vastly higher than in Figure 8(c), the stages of the event are better identified in Figure 8(c) than in Figure 8(a). The proximity to the source of vibration (debris flow) results in this high amplitude signal measured at the geophone (which is buried only at 0.5 m in depth) and difficults the distinction of increments/decrements in ground velocity.

Figure 8(a,b) corresponds to the geophone buried in the channel bed (geoCD27DIG). The IS corresponding to this geophone shows elevated values (Figure 8(b)), when using a threshold of 0.71 mm/s. The distinction between phases of the event in geoCD27DIG is plainly impossible using this threshold. However, if we use a higher threshold, such as 6 mm/s, the characteristics of the flow are visible, as in the other geophones.

Figure 8(c,d) show that both GVS and IS from geoSoilDIG reveal three stages of the event: (1) The initial phase between the beginning of the recording and the pass of the flow front (between 0 and approx. 225 s). This period is characterized by background vibration produced by the flow with the lowest sediment concentration, previous to the flow front. Several separated precursory surges, indicated by small increments of the background vibration can also be identified. (2) The passing of the flow front, indicated by a high, rapid increment of the vibration, followed by a gradual decrease (from 225 until 350 s). (3) The after flow with background vibration produced again by the flow with low sediment concentration (after 350 s).

The IS time series from geoCD27IMP (Figure 8(e)) also depicts the three phases mentioned before: the transition between the hyperconcentrated flow and the initiation of the debris flow (from 0 to 225 approximately), the flow front pass (between 225 and 300 s), and the after flow (after 300 s). However, the precursory surges before the flow front are not evident, and the background noise is lower than the one measured by geoSoilDIG. The differences in the signal measured at the three geophones (geoCD27DIG, geoSoilDIG, geoCD27IMP) can be explained by the differences in factors discussed in Section 2.2.

Thus, both GVS and IS time series are suitable to detect the phases and characteristics of the debris flow. The main difference between methodologies is that the impulses method removes the seismic noise. This noise removal is done by means of the threshold in the signal conditioner. Once again, it is important to remark the importance of the threshold, which has to be adjusted for each geophone, depending on the site-specific effects.

Moreover, there are other important differences between methodologies. Data corresponding to GVS measured by geoCD27DIG and geoSoilDIG is recorded in a higher frequency sampling rate, resulting in a file more than 400 times larger than the IS data file (Table 2). One of the most important advantages of the impulses method is related to this data reduction and concerns the power consumption. The power consumption can be considerably lower, because a standard datalogger can be used instead of a (industrial) personal computer. However, the transformation method also presents disadvantages. The main disadvantage is that it does not provide the exact value of ground velocity, neither the frequency content of the signal, because the data are transformed into a pulse signal. This simplification obviously leads to a loss of information.

### 4.3. Influence of the Vibration Velocity Threshold

**Figure 9.**
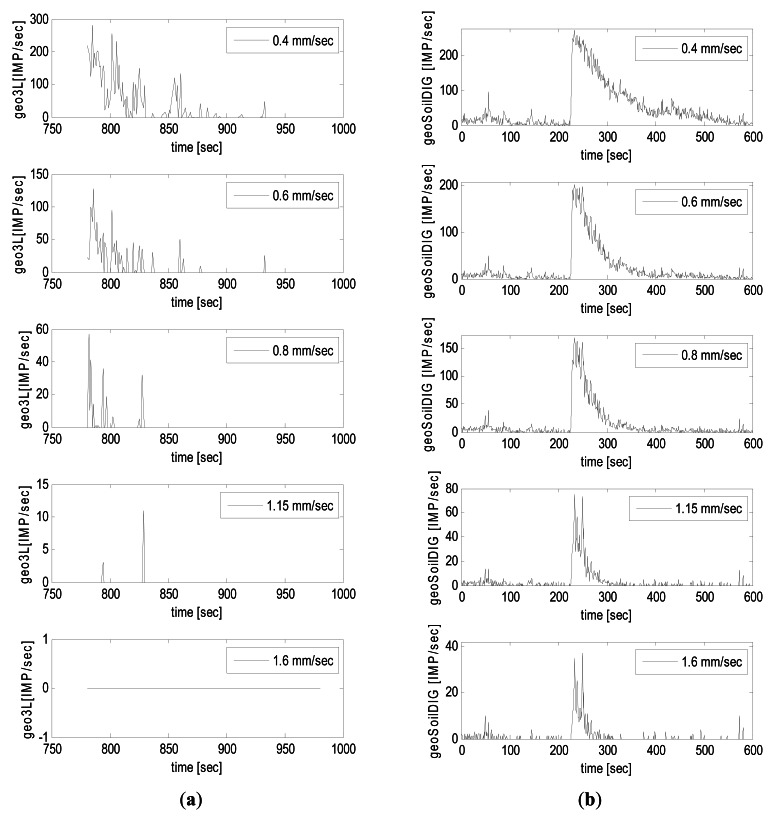
IS time series corresponding to different thresholds of ground velocity (from top to bottom: 0.4 mm/s, 0.6mm/s, 0.8 mm/s, 1.15 mm/s, 1.6 mm/s). (**a**) Dorfbach event (4 June 2011) and (**b**) Illgraben event (27 July 2009).

In order to analyze the effect of the velocity threshold value in the transformation of the GVS into impulses, the IS time series obtained from two different debris-flow events were compared using five different threshold values: (a) 0.4 mm/s, (b) 0.6 mm/s, (c) 0.8 mm/s, (d) 1.15 mm/s, and (e) 1.6 mm/s (Figure 9). The first event is the one occurred in 4 June 2011 in Dorfbach. The ground vibration signal measured at geophone Geo3L was directly transformed by the signal conditioner with the multiple thresholds indicated in Table 1. The second event is the Illgraben debris flow in July 2009 presented in the previous sections. Here, the GVS measured at geoSoilDIG was transformed by processing the data with the MATLAB code.

**Figure 10.**
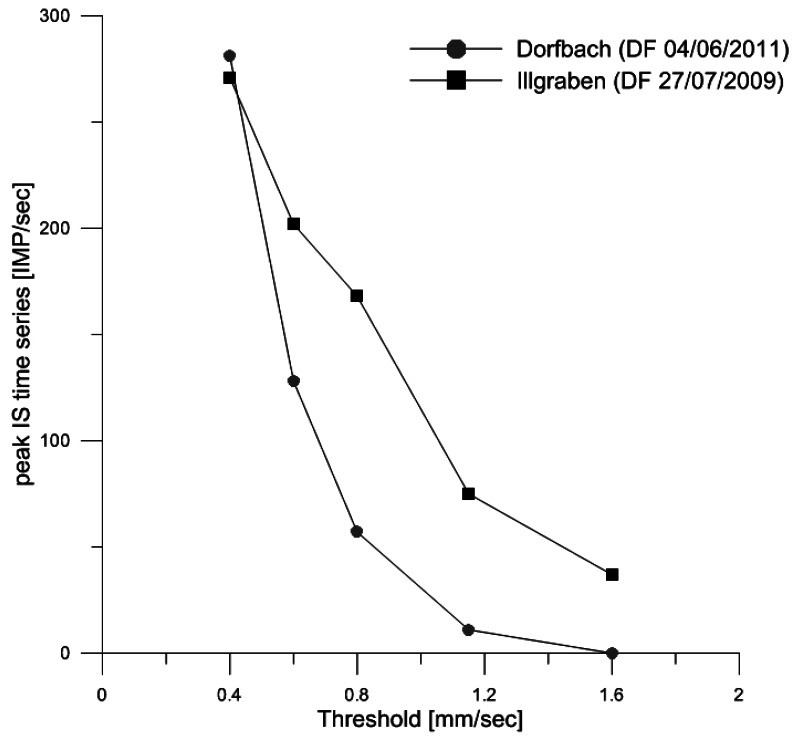
Relation between the threshold value in mm/s and the peak value of the IS time series obtained from the different cases of Figure 9.

In the Dorfbach event (Figure 9(a)), the threshold clearly influences on both the duration of the vibration and on the number of impulses. Using a threshold of 0.4 mm/s, the peak of the IS time series almost reaches 300 IMP/s and the vibration lasts 150 s approximately. As the threshold increases, the peak vibration decreases drastically (Figure 10) as well as the vibration time, until it reaches the point of no vibration, should a threshold of 1.6 mm/s be applied in the signal conditioner.

In Illgraben (Figure 9(b)), the relation between peak impulse values and threshold values is clearly visible again (Figure 10). This relation could be of great importance to detect the precursory surges, because they appear in the time series of the lowest thresholds, as they almost disappear in the higher thresholds. The time of vibration is almost not affected by the threshold, as opposed to the shape, which is actually affected. The shape of the time series and duration of the signal after the flow front has clearly a different aspect depending on the threshold value. The signal duration can be more than 400 s long in case of a 0.4 mm/s threshold, while only 50 s long if the threshold equals 1.6 mm/s.

In general, the relation of peak *vs.* threshold is visible in both events, but the signals in Dorfbach (transformed by signal conditioner) show a much higher variability (Figure 10). Thus, the results are strongly dependent on the threshold defined. There are still many uncertainties associated with the definition of the threshold. There is not yet a guideline to establish a general threshold, but nowadays it resorts to calibration or the experience of technicians and researchers. To optimize the definition of the ground velocity threshold, many aspects should be taken into account, such as ground vibration that is influenced by several factors. For example, in the two examples shown in Figure 9, we could establish that: (1) in Dorfbach the threshold should not be higher than 0.6 mm/s in Geo3L, otherwise the signal would disappear completely; (2) with a threshold up to 1.6 mm/s in geoSoilDIG, in Illgraben would still be useful to detect the signal.

### 4.4. Other Uses of the Method

Several types of mass movements have occurred at the Rebaixader monitoring station since its installation in 2009 (until January 2012). The processes monitored include two rockfalls [24], two debris flows and multiple other flows with lower sediment concentration [11]. The events were monitored by different types of sensors.

The different characteristics of rockfalls and debris flows were compared by the IS time series obtained at the four geophones. The following parameters were selected for this analysis: the peak of the IS time series [IMP/s], the mean value of IS over time [IMP/s], the sum of the seconds with IS different to null [s], the maximum slope of the IS time series within a 5 s interval [s^−1^], the slope of the IS time series within a 1 s interval [s^−1^].

**Figure 11.**
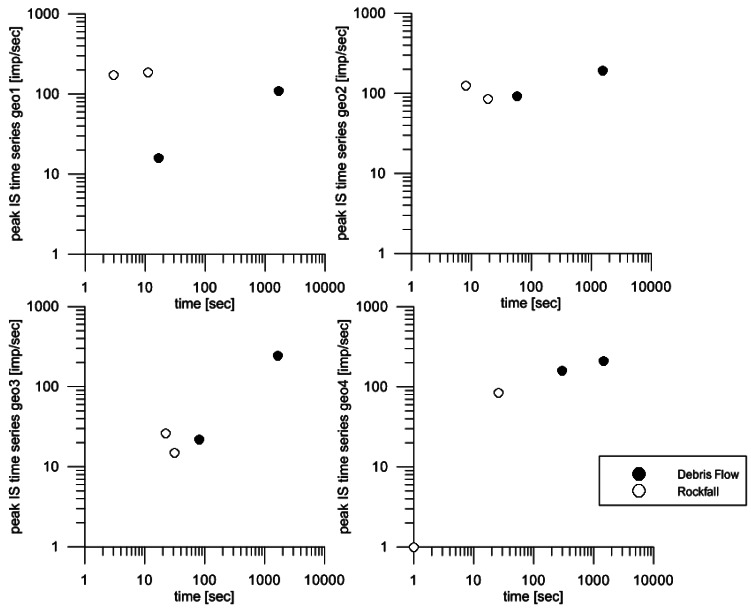
Distinction of processes in Rebaixader by using two parameters: the sum of seconds with registered impulses from the geophone and the peak value of the IS time series.

In Figure 11, the correlation between the sum of seconds with measured IS time series and the peak value of IS are shown, for the four geophones. There are two important points to be mentioned: first, at geophone geo1 (closest to the rockfall source, Figure 7(b)), the rockfalls show shorter duration and higher peak values than debris flows. The explanation is that rockfall signals are typically rapid increments of vibration generated by the impacts of the boulder detached to the ground. In contrast, debris flows produce a continuous signal with the typical characteristics of time series showed in Figure 8. Second, at geo2, geo3 and geo4 (in the channel zone, geo4 is located near the apex of the fan), the debris-flow events have longer durations and higher peak impulses. This is attributed to the fact that geophone 1 is too close to the source area and the flow is still not well developed. In all the geophones, a distinction between the processes could be established. However, geo2 and geo3 show closer signals for both processes than geo1 and geo4. This can be explained with the location of geo2 and geo3, in the middle of the travel path between the initiation area and the deposition fan. This part is where the sediment content in the flows increases the most, as well as it is a part reached by most of the rockfalls. In contrast, debris flows are not always well formed where geo1 is placed, and at the same time, some rockfalls do not reach geo4 [24].

## 5. Conclusions

In this paper, a method to transform and simplify the ground vibration velocity signal originated by debris flows and other rapid mass movements was presented. Based on the results shown, it can be concluded that:

1. In spite of not registering ground velocity signals and, consequently, losing the frequency content of the signal, the method can visualize much information concerning flow behavior (not only the flow front, but also precursory waves or secondary surges, impact of big boulders, *etc.*);
2. Due to the transformation, the seismic noise can be avoided. That is why the size of the data file can be reduced hundreds or even thousands of times. Moreover, this method reduces the problem related to the limit of power available in the monitoring stations;
3. The definition of a suitable threshold value is a key point of the method presented, in order to avoid seismic noise but to detect debris-flow occurrence. Both too high and too low thresholds can generate important lacks of information, such as missing events or losing their different stages (Figure 8(b)). It still presents many uncertainties, because the ground vibration signal is affected by many factors. That is why the threshold value of ground velocity has to be defined and calibrated for each geophone location
4. Data from the Rebaixader station pointed out interesting applications of this method to distinguish different rapid mass moving processes in a monitoring station. The results presented here, showed that the peak of the impulses per second time series [IMP/s] and the duration of the vibration [s] can be used to distinguish between debris flows and rockfalls, especially in two of the four geophones of the Rebaixader monitoring station.

## Figures and Tables

**Table 1.** Main characteristics of the location of the geophones analyzed in this work.

Catchment	Geophone code	Material in the flow path	Assembling of geophone	Distance to the flow path [m]	Threshold [mm/s]

Rebaixader	Geo1	Colluvium	Box on bedrock	25	0.17
	Geo2	Colluvium	Box on bedrock	15	0.17
	Geo3	Colluvium	Box on bedrock	20	0.17
	Geo4	Slates (bedrock)	Box on bedrock	8	0.17

Dorfbach	Geo3L	Colluvium	Box on bedrock	15	0.4; 0.6; 0.8; 1.1; 1.6; 2.3; 3.1; 4.5; 6.4; 8.9

Illgraben	geoCD27IMP	Concrete check dam	Box on check dam	5	0.71
	geoCD27DIG	Colluvium	Buried in the channel bed	0	No impulse transformation
	geoSoilDIG	Colluvium	Nailed in soil	15	No impulse transformation

**Table 2.** Details of the data recording systems of the three geophones in Illgraben with regards to the event of 27 July 2009.

Geophone code	Recorded data	Data recording system	Data recording device	File size [kB]	Number of records	Estimated power consumption of the recording device

geoCD27DIG, geoSoilDIG	Ground velocity signal	Digitization at 2 kHz	PC	114,263	1,200,000	3 A (standard embedded computer system)
geoCD27IMP	Impulses per second time series	Transformation by the signal conditioner	CR10X Campbell Datalogger	251	600	56 mA (6 mA signal conditioner + 50 mA datalogger)
